# Does Competition Work as a Motivating Factor in E-Learning? A Randomized Controlled Trial

**DOI:** 10.1371/journal.pone.0085434

**Published:** 2014-01-17

**Authors:** Bjarne Skjødt Worm, Steen Vigh Buch

**Affiliations:** 1 Department of Anesthesia and Intensive Care, Copenhagen University Hospital Bispebjerg, Denmark; 2 Department of Vascular Surgery, Rigshospitalet, Copenhagen, Denmark; University of Granada, Spain

## Abstract

**Background and Aims:**

Examinations today are often computerized and the primary motivation and curriculum is often based on the examinations. This study aims to test if competition widgets in e-learning quiz modules improve post-test and follow-up test results and self-evaluation. The secondary aim is to evaluate improvements during the training period comparing test-results and number of tests taken.

**Methods:**

Two groups were randomly assigned to either a quiz-module *with* competition widgets or a module *without*. Pre-, post- and follow up test-results were recorded. Time used within the modules was measured and students reported time studying. Students were able to choose questions from former examinations in the quiz-module.

**Results:**

Students from the competing group were significantly better at both post-and follow-up-test and had a significantly better overall learning efficiency than those from the non-competing group. They were also significantly better at guessing their post-test results.

**Conclusion:**

Quiz modules with competition widgets motivate students to become more active during the module and stimulate better total efficiency. They also generate improved self-awareness regarding post-test-results.

## Introduction

Historically, many learning methods have been used in medical education, but in recent years e-learning has been increasingly integrated along with the expansion and dissemination of digital platforms for everyday use [1]. Some of these educational applications are being developed for both pre- and postgraduate training (examples are: eFront, Moodle, Dokeos, Claroline, Ilias etc.) and used at Universities to support their curriculum.

The common definition of e-learning is rather vague. Static electronic presented textbooks, web-cam based live teaching and advanced learning games all fits the ordinary description. One should think of e-learning as a method of knowledge transfer. The *type* of e-learning can thus be differentiated into presentations, scenarios or games/simulations in the same way traditional teaching can. We have previously [2], [3] suggested a division of the multimedia *level* in order to describe how advanced the e-learning technology is.

### Student motivation

One motivation for learning is the incentive to engage in the act of gaining knowledge. Malone and Lepper [4] describe four individual motivating factors: *challenge, curiosity, control* and *fantasy*. The learning material/method should contain as much of those as possible in order to motivate the learner. They also divide interpersonal motivating factors into *cooperation*, *competition* and *recognition*.

An even bigger change may be, that the medical curriculum in many countries gradually is shifting from the acquisition of knowledge to the achievement of competence when changing teaching methods to case-based learning and problem-based learning [5]. The classic book-defined curriculum is being replaced in most Nordic countries and the students are no longer recommended certain books or learning material, but instead asked to study the relevant level.

Deci and Ryan [6] describes another theory about self-determination where *competence*, *autonomy* and *relatedness* are the key factors. When taking tests students are naturally exposed to a competence-level testing.

### Competition versus cooperation

Competition is a part of our lives. It is part of our education, our jobs and everyday life. *“It transcends time and place, as well as all manner of people”* [7]. Coakley defines competition as *“a social process that occurs when rewards are given to people on the basis of how their performances compare with the performances of others doing the same task or participating in the same event”* [8]. Graham suggests a division into direct-, indirect- and cooperative competition, and states that all holds both positive and negative components [7]. Like competition, cooperation can contain both components. Coakley defines cooperation as *“a social process through which performance is evaluated and rewarded in terms of the collective achievements of a group of people working together to reach a particular goal”* [8].

When starting studying a topic many motivating factors may be of importance. The classic incentive for studying medicine is a desire to help others, but other factors may also contribute. However, at the end of the day students will, regardless of learning method and motives, still need to pass tests and so one of the primary motivation factors may lie within these tests.

A balanced approach with both cooperation and competition could be beneficial in many cases, but obviously, learning styles are individual preferences and tendencies that can influence the learning process [9]. Overall, students learn better when subjects are presented to them in a way that matches their preferred learning style [10], [11], [12].

One way of creating a balanced project could be cooperative student teams competing against one another. Johnson & Johnson [13] agreed to the necessity to this balanced teaching method.

White and Fantone [14] argue that a pass/fail grading will reduce competition, support collaboration and foster intrinsic motivation, but many universities still use grading and there is competition to some extend. Most medical students will therefore at some point during their education have the chance to compare themselves (compete) against other students. (The aim of this study was to show weather competition could function as a motivator for better performance.).

### Games and competitions

In general games have great potential to support learning experiences. To engage in this learning process the learners needs to be motivated. According to Chan & Ahern [15], “When people are intrinsically motivated to learn, they not only learn more, they also have a more positive experience.” Quiz-modules can be set up to be relevant learning environments: they can have active experiences, and they have the capacity to provide intrinsic motivation. Intrinsic motivation relativizes the subject to the real life/practice like students often ask for.

### Motivating and ethical teaching

When motivating students to learn, competitions will, if comparison between students is made, always find winners and losers. Some may therefore argue that motivating solely by competition may be unethical.

Some evidence also suggests that cooperation increases student-efforts and creates more positive interpersonal relationships, and improves mental health compared to purely individualistic learning [16]. This argument promotes cooperation. On the other hand Dettmer [17] argued that “learning by losing” was a valuable lesson for students studying for high work-pressure jobs.

We argue that no matter what students are (at least in Denmark) in constant competition in order to get the highest grades. Though neither cooperation nor competition is solely positive or negative, this is in fact what students face.

## Aim of the Study

- This study aimed to test if competition widgets in e-learning quiz modules improved posttest and follow-up test results, self evaluation and learning efficiency.
- The secondary aim was to evaluate improvements during the training period comparing test-results and number of tests taken.

## Materials and Methods

### Entering the quiz module

This was a randomized controlled study of two groups preparing for a true-false examination. Test subjects were first semester medical students from the University of Copenhagen. Students were recruited using a Facebook advertisement (on the wall of the Danish Medical Association Copenhagen) and all interested students were invited to join. Each student was required to register and specify their gender and age. At the same time they were assigned to one of two groups using simple computerized randomization.

**Figure 1:**
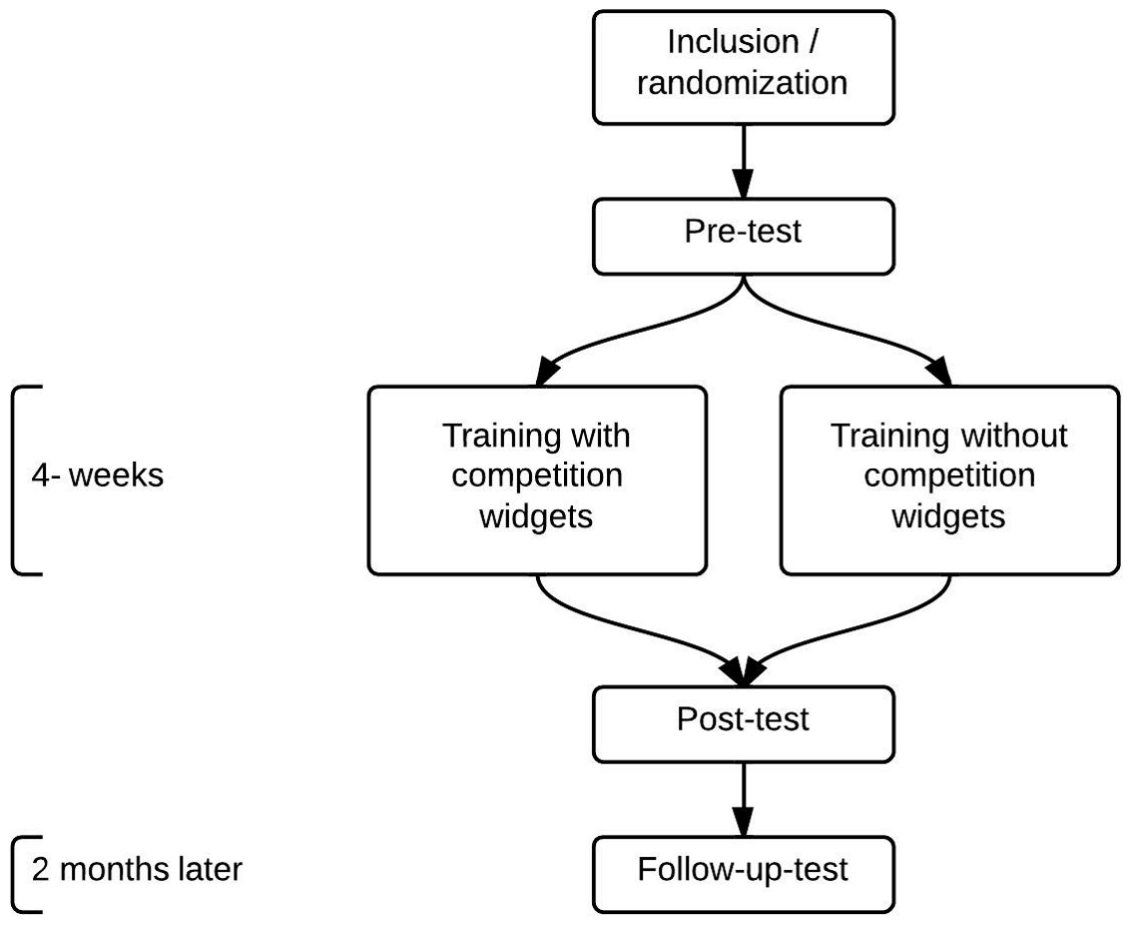
Flow sheet.

Before the students could access the module, they were required to take a pre-test. The module was available for four weeks (as demonstrated in figure 1.) and the students were asked to take a post-test followed by a follow-up test two months later. After the post-test, the students were required to specify how much time they used for studying to the exam outside the module. At a follow up conducted later the students were asked how motivated the felt during the program.

Group 1 was the non-competing group while group 2 was the competing one. In order to remain anonymous, the students were allowed to use an alias in the competition module. This option was provided in order to eliminate the risk of a lower response rate [18].

The only difference between the modules was the statistic widgets showing the student his/her score in the completed tests with the possibility to compare this to other students in the same group. The module also showed a high-score and the students could send internal mails.

### Quiz module

**Figure 2:**
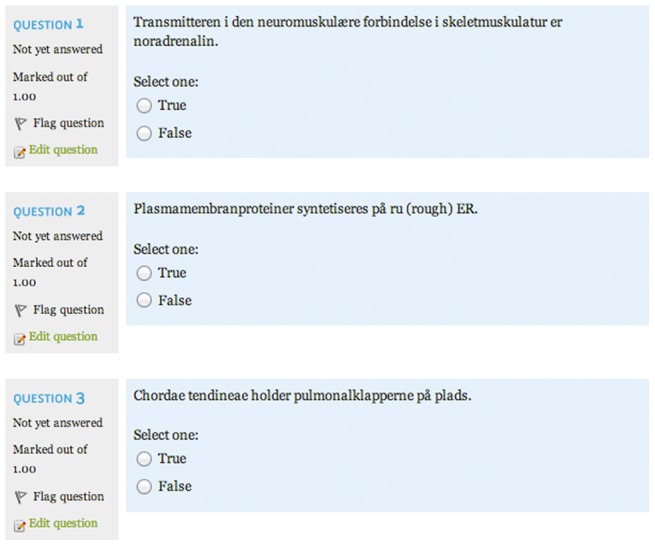
Examples from quiz module.

In the quiz module, the students were able to answer questions from a pool of 1800 different questions regarding human biology (anatomy, physiology, immunology and histology). All questions had previously been used in first semester examinations at University of Copenhagen (Faculty of Medicine). All questions could be answered with true, false or not at all (figure 2). One point was given for the correct answer, none if not answered and one point was subtracted if wrong. All tests consisted of 50 questions randomly chosen from the pool. In the training module questions were shuffled. This was done because of the large number of questions in order to compare group improvement from quiz to quiz without the bias from different predefined quizzes. In order to avoid false time registrations, students were auto-logged out after five minutes without activity. The system was configured to delayed feedback in order to motivate the students to focus on questions they had problems answering.

### Development of the test instrument

In order to avoid questions being seen before the tests, the pre-, post-tests and follow-up-test all consisted of 50 questions, randomly chosen from a special pool of 150 questions. These were validated using two groups of 25 students in pilot studies. An index of reliability was computed as the difference between the proportions of high and low scores answered correctly. All questions had a reliability index above 0.10 which was why no changes were made.

### Module development

The pre-test, post-test, follow-up-test, questionnaire and e-learning training- test-module were all designed using Moodle – a freeware (GPLv3 Licenced) PHP web application for producing modular internet-based courses integrated into a free Danish homepage for medical education. All parts of the study were closed and required password for admittance. Moodle recorded study time, number of questions tried, logins etc. The program was carried out in Danish.

### Outcomes

The primary outcome was the difference in the students' scores between the two modules obtained on the validated knowledge test.

### Statistical tests and study size

The Mann-Whitney U-test was used to compare the two groups. Cohen's *d* was calculated as a standardized measure of effect; ratio was calculated as the ratio of points (knowledge tests)/study time; ratio of points (knowledge tests)/number of tests taken and ratio of students' expectance/points (knowledge test).

Deriving experiences from a pilot study, the difference was anticipated to be 5%. Anticipated range for this difference would be 10%, thereby applying a standard deviation for all groups of 5%. We anticipated that both groups would only deviate slightly from each other, and a 5% difference was chosen as a MIREDIF. Significance level was set at 5%, and statistical power at 80%. This yielded a total requirement of 32 subjects in each group.

### Ethics

The study was purely educational and the Danish National Committee on Health Research Ethics (DNVK), Regional Region was consulted. Their conclusion was that the study did not require ethical approval (h-4-2013-fsp 41). All recruited students were asked to give an electronic consent before entering the system.

## Results

Table 1 illustrates characteristics of the 121 students who completed the pre-test, the 118 students who also concluded the post-test and the 96 students who concluded the follow-up-test. There were no significant differences between the groups regarding the demographic parameters. Table 2 illustrates differences in test score (knowledge tests), time spent with the test-training module and learning efficiency between the two groups.

**Table 1:** Demographic characteristics of participating students.

	No competition	%	Competition	%	p
*Pretest*					
N = 121	61	50	60	49.6	
Mean age (SD)	19.8 (1.0)		19.9 (1.1)		0.66
Gender					
M	24	39	24	40	0.95
F	37	61	36	60	
*Posttest*					
N = 118	60		58		
Mean age (SD)	19.8 (1.0)		19.9 (1.1)		0.54
Gender					
M	23	38	24	41	0.78
F	37	62	34	59	
*Follow-up-test and Questionnaire*					
N = 106	49		47		
Mean age (SD)	19.7 (0.9)		19.9 (1.0)		0.50
Gender					
M	18	37	19	40	0.76
F	31	63	28	60	

p values are for differences between the two randomized groups.

**Table 2:** Differences in mean numbers of correct responses, time and efficiency.

	No competition	Competition	Diff.	p	Cohen's d
*Pretest*					
N	61	60			
Mean score (SD)	15.0 (1.13)	14.8 (1.08)	0.2	0.39	0.18
*Training*					
Mean hours in module (SD)	11.3 (1.1)	12.7 (1.1)	−1.4	<0.001	1.26
Mean hours studying (SD)	122.8 (5.5)	117.2 (6.1)	5.6	<0.001	0.95
Mean examinations tried (SD)	18.0 (2.8)	33.4 (2.9)	−15.4	<0.001	−5.4
*Posttest*					
N	60	58			
Mean score (SD)	41.9 (2.84)	46.0 (2.78)	−4.1	<0.001	−1.46
Own expectance (SD)	45.5 (2.14)	45.9 (2.20)	−0.4	0.21	−0.18
Score/expectance (SD)	3.6 (3.30)	−0.02 (2.23)	3.6	<0.001	1.29
Mean improvement (SD)	26.9 (2.80)	31.2 (2.69)	−4.3	<0.001	−1.57
Mean improvement/hour (SD)	2.4 (0.35)	2.5 (0.31)	−0.1	0.26	0.21
Mean improvement/hour total (SD)	0.20 (0.02)	0.24 (0.03)	−0.04	<0.001	1.6
Mean improvement/test (SD)	1.5 (0.22)	0.9 (0.12)	0.6	<0.001	3.4
*Follow-up-test*					
N	49	47			
Mean score (SD)	39.8 (2.48)	42.4 (1.78)	−2.6	<0.001	−1.2
Mean improvement/hour (SD)	2.17 (0.27)	2.23 (0.30)	−0.6	0.38	0.19
Mean improvement/hour total (SD)	0.18 (0.02)	0.21 (0.02)	−0.03	<0.001	1.44
Mean improvement/test (SD)	1.4 (0.22)	0.8 (0.10)	0.6	<0.001	3.5

Cohen's effect size value (*d*): high (>0.8), moderate (0.5–0.8) or small (0.2–0.5) practical significance.

In the pre-test, there was no difference between the groups. The group whose module included competition used significantly more time in the training module and took significantly more tests. In both the post-test and the follow-up-test, they scored significantly (p<0.001) higher than the group without competition. The same was true when only looking at improvement (post-test – pre-test). The students competing were significantly better at predicting their own score.

**Figure 3:**
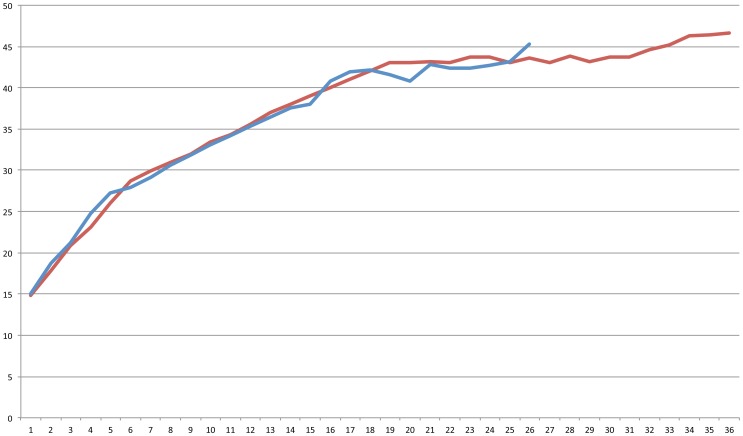
Mean training-test results depending of number of quiz taken.

Figure 3 illustrates the development in correct responses compared to number of tests taken. The line represents the mean (beginning with all students and decreasing until only one student). After about 17 tests, the improvement rate lowers. There is no obvious difference between the two groups until then.

The follow-up-questionnaire (table 3) shows an equal high satisfaction with the relevance and enjoyment of the training module. The competition group felt a significantly higher motivation (p<0.001) and they both wanted to be better than average (p<0.001) and their study groups (p<0.001) compared to the non-competing group.

**Table 3:** Follow up questionnaire.

	No competition	Competition	Diff.	p	Cohen's d
	49	47			
How relevant was the training module? (SD)	9.9 (0.3)	9.9 (0.3)	0	1.0	−0.01
How motivated did you feel during the training module? (SD)	7.0 (1.0)	8.6 (0.7)	−2.6	<0.001	1.94
Did you feel motivated to be better than average? (SD)	6.9 (1.0)	8.0 (0.6)	−1.1	<0.001	1.35
Did you feel motivated to be better than your friends or study group? (SD)	5.2 (0.6)	9.1 (0.8)	−3.9	<0.001	5.53
Do you like this kind of challenge? (SD)	9.6 (0.5)	9.6 (0.5)	0	0.56	0.12

Questions rated from 0–10 where 10 is highest. Cohen's effect size value (*d*): high (>0.8), moderate (0.5–0.8) or small (0.2–0.5) practical significance.

## Discussion

This study demonstrates that competition widgets in e-learning modules can be an important factor for student motivation. The two groups were comparable regarding demographic characteristics and in pre-test results. We find, however, that the competing group used significantly longer time in the training module and took significantly more tests than the non-competing group did.

We know that game-based learning is more effective for student enjoyment but less effective for learning [19]. However the students from the competing group reported to use significantly shorter time studying for the exam outside the system. In this study, the enjoyment therefore leads to more quiz answering and lower total studying time. The only difference between the modules was the statistic widgets showing the student his/her score in the completed tests with a possibility to compare this to other students in the same group suggesting that this *challenge* motivated the students. The follow-up questionnaire supports this finding and primarily suggests a positive attitude towards this kind of competition. An interesting fact is that the competing group both wanted to be better than average and their study group compared to the non-competing group. This might suggest intergroup competition. Students also mentioned that they wanted a possibility to be able to “directly challenge” their study group or select themselves who would be in their “competitor” group.

The competition widget proved to have a positive impact on the post-test and follow up-test scores, as well as student motivation. However, if the motivation was merely for gaining a high score and outperforming other students, some would argue that it is unethical [20]. Nevertheless, we believe that competition and outperforming others is a part of both studying and working life, thus making competition a relevant motivational factor.

From the students' perspective, it is rational to prioritize quiz answering because more quiz answering leads to better results. However, if we look at figure 3 there is a tendency towards diminishing returns after 17 tests. We expect to find similar trends in most quiz-modules, though how fast the learning curve will flatten will likely differ.

Malone and Lepper [2] state that motivators are individual and that “optimal learning environments are those that can accommodate these individual differences and the varying states of a learner's growth”. Therefore more advanced levels within quiz modules could probably limit this finding. Some of this finding may also be due to an improved ability to answer quiz questions in general.

Students from the competing group were significantly better at predicting their post-test score. The more extensive test-training may be the reason for this. One could argue that these students had a more realistic view of their own level and competences.

Our application of Malone and Leppers [2] definition of interpersonal motivating factors (*cooperation*, *competition* and *recognition*) to students studying for an online test using training quizzes was aimed to examine the competition part. This study demonstrates that competition during training have statistically significant effect on post-test results. However, at this point it is hard to tell if there is in fact an increase in competition at med-schools and it is hard to generalize across countries. Therefore, we believe that the benefits of competitive electronic learning environments should be further examined. In addition, it would be interesting to compare this effect to a group doing cooperation.

### Limitations

One could also argue that we are “teaching to the test”, but in this study we are actually seeking the improvement and also acknowledging that this is what the students were in fact doing.

We chose to use true/false questions, but there are doubts on the validity of this test type. We know that student scores are influenced by exam techniques and the willingness to take risks [21]. We also know that there may be a gender bias when answering these questions [22]. The reason for choosing this type of test was that these tests are those actually used in Denmark.

The motivating factor of competition may vary depending on ethnicity, society, age and other factors. Thus, the findings may differ in another setting and could be hard to reproduce.

### Conclusions

Quiz modules with competition widgets motivate students to spend more time during the module and yield better results. They also generate better self-awareness of their professional level.
